# Editorial: Sentinel lymph node mapping: current applications and future perspectives

**DOI:** 10.3389/fmed.2024.1422549

**Published:** 2024-05-21

**Authors:** Ramin Sadeghi, Hamid Reza Ghorbani, Malihe Hasanzadeh

**Affiliations:** ^1^Nuclear Medicine Department, Nuclear Medicine Research Center, Mashhad University of Medical Sciences, Mashhad, Iran; ^2^Urology Department, Kidney Transplantation Complications Research Center, Mashhad University of Medical Sciences, Mashhad, Iran; ^3^Gynecology Oncology Department, Women's Health Research Center, Mashhad University of Medical Sciences, Mashhad, Iran

**Keywords:** nuclear medicine, lymphoscintigraphy, sentinel node, oncology, lymphatic mapping

Sentinel lymph node biopsy is considered as one of the most important progresses in surgical oncology. In this method, the first draining lymph node of a solid tumor [sentinel node (SN)] is harvested and used as a surrogate for the involvement of lymphatic basins (1).

SN biopsy can be used for several purposes. The first is to decrease the morbidity of surgery by omitting lymph node dissection in patients with pathologically not-involved SN (such as breast cancer and melanoma). The second purpose is upstaging in tumors with very low probability of lymph node involvement which do not need staging lymph node dissection (such as endometrial cancer). The third purpose is better staging of basins by step sectioning of the SNs in cancers with very large numbers of harvested nodes (such as colon cancer) (2).

The reporting of sentinel node mapping studies is a real challenge in medical literature. The following should be reported in detail (3–7):

1. Included patients should ideally be those with no clinically involved nodes or previous manipulation of the basin.
2. Methods of lymphatic mapping should be mentioned in detail (Radiotracers, blue dye, fluorescent dyes, magnetic particles, carbon particles, etc.). All imaging techniques such as lymphoscintigraphy, intraoperative gamma camera, etc. should be reported.
3. Criteria of being SN(s) should be clear. For example, at *in vivo* count of at least five times higher than background. The method of intraoperative sentinel node detection should be mentioned (gamma probe, intraoperative gamma camera).
4. Indices of success should be clearly defined, for example detection rate as the number of patients with harvested SN to all patients. False negative rate is the number of patients with involved basins despite pathologically non-involved SN to all patients with involved basins with successful mapping. The gold standard of basin involvement can be lymph node dissection or follow up of the basins. In some cancers (such as colon cancer), upstaging following SN mapping should be reported. Any SN found outside the usual lymph node dissection should be reported.
5. In midline tumors (penile, endometrial, cervical, vulvar, etc.), each side of the patients should be considered as a separate patient and side-specific results should be reported.
6. Any effect on prognosis or decrease in morbidity attributable to SN mapping should be reported.
7. Complications related to SN mapping should be mentioned.
8. Some studies report concordance rates between two different techniques. Concordance rate, the basin which is used for concordance evaluation, and details of techniques should be clarified. Concordance studies are usually done in order to find more convenient techniques compared to the standard one.

SN mapping in gynecological cancers is an exciting frontier in gynecology oncology (8–11). It has already been incorporated in clinical practice for endometrial, cervical, and vulvar cancers. Wang et al., in a very comprehensive review, summarized the application of SN mapping in gynecology oncology. Indications, methods, guideline recommendations, and future perspectives have all been concisely summarized. Concept of SN mapping has also been used for ovarian cancers; however, it has not been addressed enough in the literature. Some studies have shown conflicting results on SN mapping in ovarian cancer: Ataei Nakaei et al. in a systematic review summarized these studies with high pooled detection and sensitivity, which showed a bright prospect for SN mapping in ovarian cancers.

Urological cancers are another target for SN mapping; penile cancer was in fact one of the first cancers in which SN mapping was validated (12). SN mapping in penile cancer is the standard of care and is done with radiotracers and/or blue dyes. New mapping materials with lower costs or less radiation have been studied, with variable results (13). Liu et al. reported their experience on the use of carbon nanoparticle suspensions in penile cancers with very promising results, especially with injection 0.5 h before surgery. Various studies reported SN mapping results in bladder, renal, testicular, and prostate cancers, and this is an area of active research (14–16). With the advent of Ga-68 PSMA imaging in prostate cancer, a new era has started in pre-operative staging of this tumor. However, lymph node staging remains a problem in prostate cancer. Fumadó et al., reported their experience in SN mapping of prostate cancers. They specifically injected the mapping material into the index lesion of the prostate. They reported 32.6% of the harvested SN outside the extended lymph node dissection, which is an important finding. A high sensitivity of 94.4% in this study is very promising.

SN mapping in thyroid cancers has been studied before, with a majority of the literature supporting its use (17–19). Boschin et al., in a comprehensive review, summarized different aspects of SN mapping in thyroid cancers. They discussed mapping materials (radiotracers, dyes, and nanoparticles), indications in papillary thyroid cancers, and advantages and limits by reviewing some meta-analyses in this regard. They concluded that SN mapping may be of value for determination of lateral neck lymph node status in thyroid cancers. Puccini et al., in another study, reviewed the available literature on SN mapping in medullary thyroid cancers. Lymph node management of medullary thyroid cancers is a matter of debate and very few studies have addressed the use of SN mapping in this clinical scenario. They discussed the results of these studies in detail and find SN mapping to be a promising method for lymph node staging of medullary thyroid cancer. Their study is particularly of interest as medullary thyroid cancer is rare and its management is under debate.

The SN concept is ubiquitous in solid tumors in all animals. Several studies have shown the application of SN mapping in veterinary medicine to decrease the morbidity of some solid tumor management in animals (20–22). Oblak et al., in an excellent review, discussed the SN concept application in veterinary medicine with special attention to cats and dogs. They introduced various mapping materials including radiotracers and blue dyes for SN concept in animals and provided several examples in real pets. Overall, SN mapping seems to be very promising in veterinary surgical oncology.

## Author contributions

RS: Writing—original draft, Writing—review & editing. HG: Writing—original draft, Writing—review & editing. MH: Writing—original draft, Writing—review & editing.
